# Islamic jurisprudential and ethical considerations of practicing medical procedures on nearly dead patients: Part I (The theoretical section)

**Published:** 2018-12-16

**Authors:** Nazafarin Ghasemzadeh, Fariba Asghari, Mandana Shirazi, Fatemeh Faramarzi Razini, Bagher Larijani

**Affiliations:** 1 *PhD Candidate in Medical Ethics, Medical Ethics and History of Medicine Research Center, Tehran University of Medical Sciences, Tehran, Iran. *; 2 *Associate Professor, Medical Ethics and History of Medicine Research Center, Tehran University of Medical Sciences, Tehran, Iran.*; 3 *Associate Professor, Department of Medical Education, School of Medicine, Tehran University of Medical Sciences, Tehran, Iran.*; 4 *Mentor, Department of Jurisprudence and Islamic Law, Urmia University, Urmia, Iran.*; 5 *Professor, Endocrinology and Metabolism Research Center, Endocrinology and Metabolism Clinical Sciences Institute, Tehran, Iran; Medical Ethics and History of Medicine Research Center, Tehran University of Medical Sciences, Tehran, Iran.*

**Keywords:** *Medical training*, *Dying patient*, *Death agony*, *Islamic jurisprudential rules*

## Abstract

End-of-life care and protection of the patient in the near-death moments are part of a patient’s rights and the duties of the medical staff. As the beginning and end of human life are most sensitive moments, there are various religious rules associated with them. The ethical issues regarding practicing medical procedures on nearly dead patients are of particular complexity and are consistent with invaluable and profoundly religious recommendations. In addition, the purpose of medical training is to provide physicians with the knowledge and skills necessary to practice appropriately and within legal and ethical frameworks. Therefore, respecting patients’ cultural and religious beliefs is an ethically accepted principle in the health systems of different countries and is the basis of respect for human dignity. The present study used a qualitative content analysis to explain how to practice medical procedures on a dying or nearly dead patient in accordance with Islamic jurisprudential rules. It was finally concluded that according to the Islamic jurisprudential rules of “authority”, “no harm”, “necessity”, and “public interest”, procedures performed on a dying patient could be used for training purposes under certain circumstances. Nevertheless, such activities should only be done with the patient’s permission and provided they cause no unnecessary harassment, and they may take place in the absence of alternative methods.

## Introduction

In addition to providing health services to people, medical science universities have an important role in training skilled human resources and specialists required in different sectors of the society. Therefore, medical training must be continuously reviewed and upgraded by eliminating the deficiencies (1). The process of medical training is influenced by several factors and variables including students, professors, educational arenas, training procedures and training resources (1). Performing clinical procedures on a patient who is clinically dead (newly dead, while the corpse is still warm) for training purposes has a long history and is common practice in many medical education centers. These procedures can be invasive or non-invasive. Endotracheal intubation or central venous catheter insertion (CV line) are among procedures that are not entirely learnable by other devices. Nowadays, alternative methods such as simulators and computer simulations are used in skill labs to train students in the abovementioned procedures, but the issues are still of great importance in discussions (2). Traditional training activities commonly performed on dead or nearly dead patients include endotracheal intubation and central venous catheter insertion, thoracocentesis, pericardiosis, placement of pulmonary artery catheter, insertion of transient intravenous pacemaker, and peripheral vascular catheter. It is clear that some physicians assume to have prerogative to use these patients’ bodies for training purposes (3). 

The most serious moral challenges in this type of training are manifested in dying patients. People experience mysterious moments throughout their lives, and the beginning and end of life are among the most sensitive and important times. These two points, the latter in particular, enjoy various rules in the form of customs and religious ceremonies (4). Since Islamic teachings have emphasized respect for the dignity of the dying person (5), moral considerations are very important at the time of patients’ death, because their dignity must absolutely be respected. At the same time, respect for patients’ cultural and religious beliefs is an ethical principle accepted in the health systems of different countries. To carefully investigate this important issue, the present study provides a comprehensive perspective on death agony in Islamic teachings and the use of nearly dead patients for medical training, and explores the Islamic jurisprudential rules in this respect. 

## Method

This research study consists of both theoretical (part 1) and field (part 2) sections. In the theoretical part, extracts were obtained from related contents in the search engines of Noormags, Magiran, Google Scholar, and SID by reviewing articles and using keywords such as “dying patient”, “death”, “Islamic teachings”, “juridical rules”, “medical training”, and “ethical considerations”. The data were supplemented through an investigation of Islamic articles and texts, jurisprudential books, and Ahl al-Bayt (PBUH) and Ganjineh-ye Ravayat-e Noor comprehensive jurisprudence softwares. The contents of the aforementioned data were also analyzed using a descriptive approach. The content analysis used in this method was conceptual analysis, that is, the components of the scriptures were dissociated and the relationship between the Quranic texts was explored. In fact, through analyzing the content of the concepts, the semantic relations between words were investigated in terms of meaning, synonymy, and semantic comparison using the principles of linguistic semantics. As a result, synonymous, opposite, general, particular, primary and secondary, convergent and divergent, single focal and multi-focal concepts and terms were distinguished (6). 

## Results

**The concept of death agony**

*Ehtizar* (death agony, dying stage, or dying) comes from the root of *Hazar*, which means “to be present”. The term along with the word “death” (*mawt*) appears in four verses of the Holy Quran. According to the encyclopedia of Islamic teachings, a person who is dying and is overwhelmed with death is referred to as *mohtazar* (moribund) (7). In other words, the term refers to a person who is on the verge of dying (8). Bahrani states, “This moment is called "*ehtizar* (dying) because of the presence of angels or family members around a dying person, or believers who are arranging a ceremony and turning the person toward the Qiblah (the point to which all the Muslims face at prayer, that is, Mecca)” (9). In this regard, Saheb Javaher also states, “Because the dying patient is close to death or because of the presence of the angels and the Imams (PBUT), or the presence of believers at the patient's bedside for the purpose of planning a funeral, or a wise authority, or for all of these reasons, these moments are called *ehtizar* (dying)” (10). Also, it is said that consciousness and the presence of the mind is very intense in the dying moments (11).

1- Al-Baqarah, verses 133 and 180; Al-Nisaa, verse 18; & Ma'ida, verse 106.

**Dying in Islamic texts**

The Quran has addressed the issue of dying but has not used the term “*ehtizar*”. Instead, it has used the following terms and expressions:* “sakr al-mawt*” (agony of death); *ghamaratu al-mawt* (the pangs of death); the presence of death; the coming of death; the soul of a dying person cometh up to his throat; and *tawwaffa*, which means the taking and keeping of the soul. Therefore, expiring or dying in Quran and the traditions of Ahl al-Bayt (PBUT) are also called moments of *sakrat *(agony) and *ghamarat* (pangs). Given the lack of a clear definition for death in the Holy Quran, and despite the assumptions made about the indicators of death in the traditions and practices of Prophet Muhammad, the task of defining death cannot be assigned to the existing literature, because they describe death using incorrect terms (13). For this purpose, we examined Islamic sources to find the signs of death. The Quran has indicated some signs for the dying person. In verses related to the Battle of the Parties (*Ahzab*), when the fear of failure appears in a group of saboteurs, they are described as being surrounded by death; they are distressed and their minds start to decay. “As the fear comes, you will see them in the throes of death, their eyes revolving in their sockets” (14). There are also signs of being on the brink of death in *hadiths* (a collection of traditions containing the sayings of the prophets). Imam Baqer (PBUH) said, “When dying, the believer’s face goes pale, it turns whiter than any other part of his body, his brow is covered with sweat, and tears fall from his eyes; these are the signs that show the soul is leaving the body” (11). By these descriptions, it is not difficult to identify the dying person (*mohtazar*) and recognize the signs, although it is not impossible to be mistaken either. In juridical texts, there are recommendations regarding dying persons in the form of religious duties that are obligatory (*wajebat*); recommended acts, which are desirable and rewarding but not mandatory (*mustahabat*); and detestable acts, which should best be avoided though not absolutely unlawful (*makruhat*). One of the most common recommendations is to help the dying person face the *Qiblah* (15-17), which is obligatory for every Muslim and for which the guardian’s permission is not necessary (18, 19). It is also advisable to read the Surahs Yasin and Saffat at the dying person’s bedside (20). In addition, placing heavy objects on their abdomen or talking endlessly in their presence is to be avoided (*makruh*) (18, 21, 22). 

**Importance of medical training and the challenges of the use of nearly dead patients**

In the field of medical education, professional and clinical skills cannot be learned solely through reading books and attending lectures, but need to be acquired in real-life situations and environments (23, 24). Therefore, it is essential to use patients for the purpose of medical training, as they have always been involved in medical education (25-27). In terms of clinical training, dying patients have long been the focus of educational programs, and have therefore received careful considerations. Patients who are in their death throes have two educational advantages over newly dead patients. Firstly, they create a real environment for practicing procedures such as cardiopulmonary resuscitation (CPR) as, for instance, endotracheal intubation and central venous catheter insertion are more difficult to perform on these patients during chest compressions. The second advantage is related to procedures in which physiological responses are a sign of technical success, as these signs are absent in the dead person. Examples include fluid return during lumbar puncture and blood return during insertion of the central venous catheter (3). Although respect for the dying person has been emphasized in Islamic culture (5) and certain rules exist in this regard, serious ethical challenges arise occasionally due to the delicacy and inseparability of medical treatment and educational activities. As an example, resuscitation is a procedure that is not discernible for the families, and may therefore be unethically extend by physicians in order to create further training opportunities for trainees. Nevertheless, resuscitative procedures are recommended merely for medical purposes so as not to cause further pain and suffering for the patient. When performed for educational purposes, resuscitative activities may prevent immediate death and thus prolong the dying process, or aggravate the patient’s condition and lead to

 disabilities resulting from the inefficiency of the trainee as well as unfavorable conditions (3). Physicians assume that the significance and benefits of such educational procedures are far greater than, their potential physical harms. It should be noted, however, that there are other considerations in this regard, including the social expectations for proper behavior and treatment of dying patients and newly dead corpses, the demand for these patients to benefit from appropriate care, and the health and welfare of future patients (3).

1- Ghaf , verse 19.

2- An’am, verse 93.

3- Nisaa, verse 18.

4- An’am, verse 61.

5- Waqi`ah, verse 83.

6- Nahl, verse 32.

7- ‘*Ghamarat*’ is the plural form of ‘*ghamre*’ meaning cyclones and hardships. In fact, at the time of near death, man undergoes a sort of senselessness in which the whole world is seen as darkness and there is no sensation except excruciating pain (12). 

8- Ahzab, verse 19.

 On the other hand, dying patients should be regarded as living persons and it is necessary that the procedures on these patients be based on medical necessity and compassionate care. Essential medical procedures should be performed on everyone without discrimination, and should therefore be practiced for dying patients and logged in their medical records on a regular basis. Moreover, all procedures performed by trainees should be carefully monitored by qualified physicians. Medical staff and health-care providers should respect the dignity of each patient until death, and should continue to respect the corpse afterwards. It should also be noted that training activities should not interfere with the patient’s family visits and must have no effects on the results of an autopsy or evidence of forensic medicine (28, 29). 

On the one hand, in order to promote the educational system and improve learners’ skills especially in life support procedures, using dying patients for training purposes seems inevitable, since artificial substitutes will not be as effective. On the other hand, patients have the right to receive the best physical care and emotional support possible, and their dignity should be protected in all situations. 

**Respect for human dignity**

Philologically, respect (*ehtiram*) means having reverence, or being honored (30), and dignity (*karamat*) means greatness or being venerable (31). Dignity can be examined at least in three areas: human dignity, divine dignity, and conventional/social dignity (32). Therefore, human dignity is considered to be one of the fundamental rights and the basis for other rights (33) because dignity means to value humanity and requires that individuals enjoy their innate, natural and social rights including the right to live, the right to freedom of thought, and so on (33). However, dignity is not considered a right in the strict sense of the word, although it is the intrinsic identity and innate nature of humans. Therefore, dignity is not separable from humanity, and for this reason, all individuals, regardless of their opinions, beliefs and religions, are entitled to dignity (34). According to the Universal Declaration of Human Rights, dignity is based on human rights as a principle, that is, mankind is human and merely because of that, he/she has certain rights. Accordingly, no external issue can take away a person’s rights and his/her honor or humanity, and dignity should be protected in any event (35). Therefore, since humans are endowed with human nature and intrinsic faith as one of its components, they have dignity and they are bound to protect it. Moral and legal rules also help individuals honor that most valuable essence of self-worth (36). It is noteworthy that in religious texts and sources, there are two kinds of dignity: intrinsic dignity and acquired dignity. This categorization in its essence represents the comprehensiveness of the religious perspective on this human attribute, which has not been extensively taken into account in non-religious legal sources.

*A. Intrinsic dignity*

Dignity is inherent in all human beings from the first days of their lives as well as the onset of their individual development, and will remain an intrinsic part of everyone’s existence until they deprive themselves of its merits (37). In other words, humans enjoy such an attribute as long as they do not reject it through committing a crime or betraying themselves and others by their own will (38). According to various legal schools of thought, both divine and non-divine, the inherent dignity of humans is the basis for the emergence of different rights for human beings, although each school regards human beings according to its particular worldview and analyzes them comprehensively or partially (33). In other words, this kind of dignity is innate, intrinsic, and unbound by religion (39). 

The verses and authentic narrations refer to this type of dignity. The root of the word *Karam* and its derivatives have been cited 47 times in different verses of the Quran (in 29 Surahs and 46 verses). Meanwhile, verse 70 of Surah al-Asra, which describes how God has honored man, is the most important evidence of human dignity: “And indeed we have honored the sons of Adam, and we mounted them [on horses] in the midst of the land and the sea, provided them with clean things (lawful edibles) and given them obvious superiority over many of our creatures” (14).

*B. Acquired dignity*

Acquired dignity, which is also called value dignity, is non-intrinsic and dependent on individuals’ opinions, and is defined in terms of man’s relation to God (39). This is the kind of dignity and honor which humans acquire voluntarily and through the use of their talents and inherent abilities in the process of growth, perfection, and acquisition of moral virtues (40). The criterion of acquired dignity is virtue. Verse 13 of Surah Hujurat points to this criterion: “O mankind, we have created you from a male and female, and made you into nations and tribes that you may know one another. Verily, the most honorable of you in the eyes of God is the believer who is the most righteous” (14). It is worth mentioning that in terms of human rights, there are different principles for human dignity in the West, so that it is based on thoughts such as humanism, natural (innate) rights, positivism, and pragmatism (41). Nevertheless, the mentioned categories are not consistent with religious thought. Consequently, from the contemporary Western perspective, universal human rights have made human dignity visible only in inherent dignity, and do not take into account the higher dignity that we call “value dignity” and thus stop the perfection of humanity in the course of reasonable life (38)**. **However, all human beings are presumed to have inherent dignity, and exercising this right leads to certain rights and duties. In medical education, this important issue is also true of a dying patient. Therefore, medical service providers should also respect the dignity of all patients until their death, and should even respect the corpse after death (28, 29). 

A detailed investigation of different schools suggests that dignity is the most important human attribute and must be preserved unconditionally. Therefore, it is essential to respect the dignity of dying patients in the course of medical training. From the point of view of Islam, dignity pertains to the human soul, and the body also follows dignity (42). Thus, even though medical education is necessary, training activities should not threaten human dignity or prolong the process of dying. In this regard, Islamic jurisprudential rules (*Qawaid Feghhi*) and the views of jurists can be used to find answers, since jurisprudential rules play a significant role in the comparison of particular cases, and examples of each of the general rulings are of particular importance. 

***Islamic jurisprudential rules***

Invoking jurisprudential rules is merely possible in the proposed discussion. The following sections will examine the most important juridical rules, namely, “the rule of *saltanat* (monarchy/authority)”, “the rule of *la-zarar *(no harm)”, “the rule of *zarurat* (necessity)”, and “the rule of *maslahat* (expediency/public interest)”.

**The rule of Authority (Saltant or Taslit)**

One of the most established rules of jurisprudence has been referred to by different names: the rule of “authority”, “dominance”, “monarchy”, “control”, and “the absolute dominion rule”, but is commonly known as “the rule of monarchy” (*taslit)* in jurisprudence. The word “*taslit*” philologically denotes that someone is dominated by another with paramount authority (43), and the concept is rooted in the prophetic *hadith* “*Al-Naso Mosallatoun Ala Amvalehem*” (People have domination over their properties) (44). The word “*Al-Nas*” refers to all people, men and women alike, because the relative pronoun “*Al*” is inclusive of both male and female genders. There are both positive and negative components in the rule, which means every owner has domination over every one of his/her properties, and this domination can be limited neither by a person nor an institution (45). 

The rule is based on the Holy Book (Quran), tradition (*sunnah*), consensus (*ijma*), and reason (*aql*). There are several verses in the Quran that indicate people have full control over their possessions and are entitled to any kind of seizure, including Surah Al-Nisaa verses 2 and 29. In this regard, Surah Al-Baqarah, verse 188 states: “And do not seize one another’s properties unjustly, and do not offer a part of it [as bribe] to the judges to help you take possession of other people’s properties sinfully while you are [well] aware of it” (14). Careful consideration of the above verses indicates that every human being has dominance over his/her property and has the right to any seizure in concordance with religious principles, and the seizure of others’ properties is subject to the consent of the owner (46). 

“People dominate their property” (44) is the most famous narrative cited by jurisprudents. In terms of documentation, the narration is weak, but its weakness is compensated by the actions of the companions of the Imamiyah (PBUH) who are all trustable witnesses and tradition narrator jurisprudents. Thus the *hadith* of *Taslit* has such great credibility that, despite the weakness of the narrative, it is considered as a jurisprudential rule (47). 

In particular cases, narrations have also been cited that imply the owner’s control over his/her property, including the following: Mohammed the Prophet (Blessings of God be upon him and his descendants) said, “A Muslim’s property is respected just like his blood” (48). Moreover, the Prophet (PBUH) says in another narrative, “It is not permissible to use a Muslim’s property except with his consent” (49). Referring to jurisprudential books, it becomes clear that all jurisprudents have reached a consensus on this rule and have referred to it in various cases (50-52). Also, examining this rule reveals its rationality, and rational scholars have acted upon it since the past until the present day. Some even believe that rather than being a rational rule, this rule is a natural one that is rooted deeply in the human soul, and because of the conformity of Sharia law with the laws of nature, Sharia has also confirmed this innate law; therefore, the content of this rule is not an Islamic order, but a natural and rational statement that Islam has also confirmed (46). Although documented evidence suggests that the rule of *taslit* pertains to property, some jurists have applied it to rights as well (53). Rights have not been mentioned in *hadiths*, but the rational idea is that every person has control over his/her property as long as the holy *Sharea* (legislator) has not issued a command otherwise. On the other hand, based on the implication of priority, people’s domination over their property should be generalized to their rights as well (46). In other words, control over one’s property can be exercised over one’s soul and rights. This is the rational rule of “People having domination over their property and souls” (54). The point is that if possession of property is contingent on consent of owners, possession of their rights and souls must also be based on their consent. Therefore, when practicing medical procedures on dying patients, their consent should be obtained in order to respect their rights (28, 55). In this case, performing certain procedures for training purposes would raise no objection on the side of the patients or their families, since they have given their consent with respect to this rule. 

In recent years, the use of the four ethical principles of autonomy, beneficence, non-maleficence and justice has become widespread in the field of biological and medical ethics in relation to human beings. These principles have their origin in the West and at times share similarities with some other concepts, creating challenges in certain areas. Autonomy is a case in point that is sometimes confused with free will, and therefore a discussion of these two categories is presented below.

**Autonomy**

Considering the large number of references to autonomy in the field of biological and medical ethics, nowadays there is a need to answer the question of what autonomy is, and to explore the basis, of autonomy as well as its similarities and differences with free will. Autonomy, which is synonymous with free will and self-government, means that individual independence is one of the external factors and rights of a person in terms of his/her personal affairs (56, 57). The concept has been influenced by Imanuel Kant, who believed that in order to clarify free will, we must understand that it has the power to create works and deeds, but is not the effect of something else (58). He considers freedom as the autonomy of will, so this will must lay down laws that individuals must obey. He defined will in the sense of free causality, according to which the effect is prompted by the cause. But the law of autonomy is fundamentally different from the natural law of cause and effect, because the will that is induced by natural necessity is not free, since the causal action is the subject of something other than the subject itself and cannot be considered freedom. In nature, the causal action of an efficient cause is brought about by something else and not spontaneity. In other words, the law governing causal action is not self-imposed, but is laid down by another, and it is called “heteronomy”. Consequently, if the law of will is to be imposed by something else, that is, it is not autonomous; then it is not compatible with the will of man (58). According to Kant, religious morality is a type of ethics in which the acts of humans are regulated by divine commandments centered on the hope for paradise or fear of hell, and is therefore part of a heteronomous ethics that Kant does not consider ethical (58). In view of his reliance on autonomy, there is no place for heteronomous ethics in Kantian philosophy. 

**Free will**

All divine laws are based on the principle of free will. Divine prophets themselves were the harbingers of free will and invited people to seek and establish freedom (59). As God says in Quran, “Then, whoever wants may believe and whoever wants may disbelieve, as we have prepared a fire for the oppressors”. Furthermore, in two verses of Quran, God has stated, “He would not change people’s destiny until they themselves change their destiny”, and “We showed him the way, whether he be thankful or ungrateful” (14). Thus, free will is the integral cause of human actions. In fact, God bestowed wisdom, free will and power upon man, giving him a position superior to other living things to be autonomous in his own acts (59). Human autonomy is the cornerstone of Islamic juridical and ethical system in defining law, duty, and responsibility. The coming of prophets and the revelation of heavenly books would clearly be in vain without the autonomy of humans. This shows that God and prophets recognize the 

1- Al-Kahf, verse 29.

2- Ar-Ra'd, verse 11; Al-Anfal, verse 53.

3- Al-Insan, verse 3.

power of free will (60). On the other hand, according to Kant, the will is to be regarded as the originator of works and actions, which should not have sources other than the individuals’ willpower. On the basis of unity of divine acts, all actions throughout the world are God’s and all acts and influences are issued through the essence of God and no subject has independence in effect (61). However, the will of God and man are dependent on one another, that is, what is done by the will of man is a result of God’s will (60). Nevertheless, since the action arises directly from man’s free will, attachment to the divine will does not lead to obligation as the arbitrary criterion of action is that it derives from free will (62). In addition, reference to the principle of “authority” in Islamic jurisprudence and Islamic law involves the ability of people and their domination over their properties and their lives, so that they are free to use them and others do not have any rights to dominate and seize them. On the other hand, Kant also believes that morality becomes meaningless if humans are not free. Respect for the principle of human being’ freedom and their inherent right requires that no actions occur against their will and desire. However, the four logical principles indicate that there is a logical relationship between autonomy and free will, that is, they do not completely conform to each other, but there are common similarities and differences between them. Freedom should be considered as a common aspect between autonomy and free will. A dying person who has allowed the medical staff to practice clinical training activities on his/her bedside (through consent or will) has decided based on his/her own free will without any obligation. 

**The rule of no harm (La-zarar)**

One of the most famous juridical rules that has been considered a significant resource in many jurisprudential issues is the “no harm” rule. This rule is so significant that for centuries, numerous jurisprudents have devoted a complete treatise to it (63), and have exponentially elaborated it. The rule of “no harm” has a strong Quranic and narrative validity as there are verses in the Quran that have made rulings by specifying the term *zarar* (harm or loss) and its derivatives in certain cases. Instances include verses 282, 231, and 233 of Baqarah, as well as verse 12 of Nisa. As an example, verse 233 of Surah Baqarah states, “No mother shall be harmed by her child, and no father shall be harmed on account of his child” (14). According to this verse, it is prohibited and illegal for anyone to harm another. This denial of what many consider to be the reality is due to reverence for human life, and does not pertain to one particular case. In other words although the verse speaks about parents, it is not limited to paternal and maternal characteristics (64), and can be extended to similar cases. There are also so many *hadiths* related to this principle that mention the same point exactly and deal with the same subject (63), and in all of them incurring loss has been forbidden. The most prominent narrative based on this rule is one that has been continuously mentioned in credible sources. According to this narrative, Prophet Muhammad (PBUH) once said, “You are a harmful man and harmfulness is not acceptable in a believer” (65, 66). The words used in the cited narratives of the abovementioned rule do not have a particularly religious meaning, but should be interpreted in their conventional sense. Thus, the term “*zarar*” refers to any defect that is inflicted on the property, body, honor and rights of individuals (67). In addition, rational thinkers’ criteria should serve as a firm foundation for this rule. Undoubtedly, wise people agree that causing somebody harm is considered an unpleasant act in social and civic life and whoever causes damages is responsible for payment and compensation, and therefore this principle has been accepted in all legal systems. Since the holy *Sharea* has imposed no prohibitions on such a rational criterion, confirmation of the holy Sharea (legislator) is taken in this respect (63) and as a result, it can be used to counteract permission that may lead to another harm (68). In the light of this rule, any training activity on a nearly dead patient is permissible only in the absence of harm to the patient, as prevention of harm is the most important issue according to the documents on the rule. The word ‘harm’ specifically includes any loss that may be inflicted on the rights of individuals. Moreover, the dying patient is not excluded from this rule, and any harm by the trainee is an encroachment on the patient’s rights, which contradicts the ‘no harm’ rule. Therefore, it is acceptable to provide compensation for the harm and trainers are liable for injuries caused by their trainees during training period.

**The rule of necessity (Zarurat)**

In Arabic terminology, “*Zarurat*” (necessity) is the noun for “*ezterar*” (urgency) (43). In common sense, necessity and urgency (*zarurat* and *ezterar*) are used together in the sense of necessity and urgency (69). Necessity may be the ground for committing certain prohibited acts (70). In Islamic law, a crime may be justifiable through concepts such as “prohibitions become permissible under necessity”, or “necessity knows no law” or “necessity precedes the law”. As a result, necessity has always been a reason for renunciation of liability throughout history (71). In addition, it is reasonable to ignore the lesser benefit in order to maintain the greater one in a state of emergency (72). The justification is that harm is permissible whenever there is a significant necessity or expediency that can be achieved through causing the harm (73). It is worth noting, however, that alongside the above rule, there is also the principle of “what is required is measured by what is required” or “there are always specified extents for necessities that should be observed accordingly”. The abovementioned principles form a valid juridical rule, that is, the secondary ruling that is due to necessity or urgency and is only limited to what is necessary. This rule prevails in legal affairs including civil responsibility (74). In other words, the scope of the necessity rule is not broad and the smallest degree of necessity should suffice. 

Various verses and narratives have confirmed the importance of necessity directly or by provision, which can be referred to as evidence for the rule. There are verses in the Holy Quran that could be mentioned as grounds for the rule of emergency. Verse 173 of Surah Al-Baqarah, for instance, states, “[Allah] has forbidden you only carrion, blood, and the flesh of swine, and that which is [slaughtered as a sacrifice] for others than Allah (or has been slaughtered for idols, etc., on which Allah’s Name has not been mentioned while slaughtering); [but] if one is forced by necessity, [one can eat these meats to save one’s life and] there will be no willful disobedience nor transgression of the due limits, and it is no sin. Truly, Allah is the Oft-Forgiving, and the Most Merciful” (14). The ordinance of the verse does not pertain to the afore-mentioned circumstances, and the case is not exclusive. If the case and the cause of revelation for this verse was limited to a particular reason, we could assume that most of the verses had been provided for a particular case or a specific cause of revelation and could not be generalized to other occasions (46). Another verse states, “Why should you not eat that upon which the name of Allah has been mentioned [at the time of slaughtering the animal], while He has explained to you in detail what has been forbidden to you, except what you have forcibly required?” (14). In the above verse, what is known in Islam as *Haram* (forbidden, prohibited, not legal or allowed by law), or is ordinarily regarded as filthy, is considered permissible in the light of a general and universal rule (urgency), and the scope extends to all *Harams*, since the exception in this verse refers to any of the *Harams *mentioned by the holy legislator. The exception, that is, the permission to commit a *haram* in an emergency, will belong in the territory of all *Haram* deeds (46), as there is no intention to commit a *haram* in the field of medical education in the case of a nearly dead patient. At the same time, the training may also help solve some of the problems of the medical community in the future.

The most important reason for the narration referring to urgency is the *Hadith* known as the *Hadith* of the *Raf'*. In this hadith the Prophet (PBUH) said, “My *ommat* (community) will not be condemned for 9 errs: mistake, forgetfulness, what they do out of necessity, what they do not know, what they are unable to do, what they are forced to do, jealousy, evil omen (auspices), and the temptation to think of creating life, as long as they are not spoken or performed” (48, 75). In addition, this *Hadith *is ordained by the holy legislator for the benefit of the community and cannot be confined to a particular person or individual (71). Medical training activities that are performed on dying patients seem to be a case in point and can be the subject of the above rule.

1- An’am, verse 119.

 **The rule of expediency (Maslahat)**

The principle of *Maslahat* (public interest) should be considered as one of the rules applicable to various affairs of the society, as well as an effective rule of jurisprudential dynamics. *Maslahat* is based on *Maf’alat* (an Arabic rhythm) and is derived from the word *solh* (peace), meaning “interest”, the opposite of which is corruption. About this word, Ibn-e-Manzour writes, “*Eslah *(improvement, reform) is the opposite of *fasad* (corruption), and *maslahat* means *eslah*” (43), and in Majma Al- Bahreyn, he also states, “…and in the affair there is interest or good” (76). In this regard, Saheb Jawaher writes, “It is inferred from the narrations and sayings of the jurisprudents as well as the Quran that all transactions and non-transactions are proclaimed for the expediency and worldly or other-worldly benefits of the people, all of which are conventionally called expediency and interest” (51). According to Allamah Heli, expedient is something that is compatible with human intentions, either worldly or other-worldly, or even both (77). All Muslims believe that God has a reason for his ways, and all the Sharia rulings have specific purposes and intentions, because otherwise, everything would be in vain (78). People’s actions are no exception to this, and there should be an underlying purpose for every action. There are some issues that the holy legislator has not addressed, delegating them to people to resolve according to the benefit of the community (79). Clinical medical training performed on a dying patient is one such case.

The term *Maslahat* does not appear in the Quran, but many derivatives of this term, such as *eslah* (their affairs, improvement), *aslah *(reform) and *saleh* (righteous), have been used dozens of times. The holy legislator urges human beings to be peacemakers and serve the benefit of the community, and observe what is considered to be public interest, in verses such as Surah A’raf, verses 85 and 170; Surah Al-Baqarah, verse 220; and Surah Al-Hud, verses 116 and 117(80). Nowadays the principle of utilitarianism has overshadowed everything, and medical education, especially when involving a dying person, is no exception, since lack of training in certain conditions may harm the success of medical education. Therefore, it is essential to allow this type of training to be provided for the benefit of the community, on the religious condition that no distress and constriction is imposed on the dying patient.

## Conclusion

By considering the Islamic jurisprudential rules mentioned earlier, it can be concluded that they can be applied when practicing medical procedures on a dying patient. In this regard, see Table 1 below.

**Table 1:** Application of Islamic jurisprudential rules in practicing medical procedures on a dying patient

**Islamic ** **Jurisprudential ** **Rules**	**Their Application in ** **Practicing Medical ** **Procedures on a Dying ** **Patient**
*Saltant* (Authority)	Patient-centeredness, informed consent
*Zarurat* (Necessity)	Necessity of medical education on dying patients in lack of alternative methods
*La-zarar* ( No Harm)	Avoidance of any harm, distress or prolongation of the dying process
*Maslahat* (Public Interest)	Community-centeredness, benefit to community

As previously pointed out, the foundations of human dignity differ in Islam and the West. In the Islamic view, the integration of soul and body has dignity and therefore the body deserves respect due to the presence of the soul. In the West, due to the intellectual foundations that are mostly rooted in the human nature, dignity is regarded rather similarly. However, the intrinsic human dignity should be considered as a common prospect in all schools of thought. Moreover, free will adds to this dignity, and at the same time entails various issues such as obtaining the consent of a dying patient for medical training as well as using his/her body for medical practices according to his/her will. Nevertheless, it has been extensively explained in this article that free will should not be considered equal to autonomy since the human will is dependent on the will of God. 

The conflict here is that on the one hand, medical training, particularly on a dying patient, is essential for the education of trainees, and on the other hand, the patient is not completely dead, and training procedures need to be practiced objectively and experimentally. Even though some forms of training could not be practiced in any other environment, Muslims’ right and dignity must be observed and respected. In such cases, one should refer to the narrative that “necessity removes the ban”, or consider the justification that the health of the community depends on these training procedures. Under the circumstances, performing training activities on dying patients is permissible, provided that the patient’s consent is obtained. However, these activities should not result in any harm or distress, and in the presence of a will (a patient’s will or advanced directive), the procedures should be performed according to it. 

## References

[1] Emami H, Aghdasi M, Asousheh A (2009). Electronic learning in medical education. Research in Medicine.

[2] Larijani B (2004). Health Care Professional and Ethical Issues. Tehran: Baraye Farda.

[3] Berger JT, Rosner F, Cassell EJ (2002). Ethics of practicing medical procedures on newly dead and nearly dead patients. J Gen Intern Med.

[4] Sadeghi H, Nosratian Ahoor M (2013). Subject analysis of death. J Med Ethics Hist Med.

[5] Heidari MR, Anoosheh M, Azad Armaki T, Mohammadi E (2011). Nurses experiences in the care of end stage patients. J Med Ethics Hist Med.

[6] Rafipoor F (2003). Special Research Techniques in Social Sciences.

[7] Hoseini SM (2010). A Dictionary of Terms in Islamic Jurisprudence / Arabic – Persian, with English Equivalents.

[8] Al-Asfahani R, Al-Husayn IM (1992). [Mufradat Alfaze al-Quran].

[9] Bahrani Y (1984). [Al-Hadaiq Al-Nazrah Fi Ahkame Al-Etrato Al-Taher].

[10] Najafi MH (Saheb Javaher) (1981). [Jawaher Al-Kalam Fi Sharh Sharaea Al-Eslam].

[11] Qomi Sadugh M (1992). [Man La Yahzaru Al-Faqhih].

[12] Ibn Manzur M [Lisan Al-Arab].

[13] (2011). Islamic Biomedical Ethics Principles and Application.

[14] Holy Quran (2009). Osveh Press.

[15] Tarabulusi A (1985). [Al-Mohazzab].

[16] Tusi M (1968). [Al-Mabsoot Fi Feghhe Al-Imamyeh].

[17] Tusi M (1979). [Al-Nihayah Fi Mojjarad Al-Fighhe Va Al-Fatawe].

[18] Husaini Seestani A Rules related to dying person.

[19] Mousavi Khoei SA (2003). [Resaleh Tozihol Masael].

[20] Helli SR (1985). [Falahol Saele Va Najah Al-Masael].

[21] Mousavi Khomeini SR (1989). [Tahrir al-Wasilah].

[22] Shubairi Zanjani SM (2008). [Al-masael Al-Shariah].

[23] Gomes AP, Rego S, Palácios M, Siqueira-Batista R (2010). Bioethical analysis of the use of newly dead patients in medical training. Revista da Associação Médica Brasileira..

[24] Draper H, Ives J, Parle J, Ross N (2008). Medical education and patients’ responsibilities: back to the future?. J Med Ethics..

[25] Jagsi R, Lehmann LS (2004). The ethics of medical education. BMJ..

[26] Howe A, Anderson J (2003). Involving patients in medical education. BMJ..

[27] Doyal L (2001). Closing the gap between professional teaching and practice: a policy can help protect students from being asked to behave unethically. BMJ..

[28] Jetley AV, Marco CA, Jesus J (2012). Practicing Medical Procedures on the Newly or Nearly Dead. Ethical Problems in Emergency Medicine: A Discussion-Based Review.

[29] Marco CA, Brennan JA, Chipman CD (2003). Teaching procedures using the newly dead. ACEP Clinical & Practice Management.

[30] Moein M (2009). A Persian Dictionary.

[31] Moein M (2009). A Persian Dictionary.

[32] Haqiqatpur H (2013). A Research concerning the principles of the right for human dignity in sources of rules. Journal of Fiqh and Usul..

[33] Montazeri HA (2014). [Resalat Al-Hoghogh Fi Al-Islam].

[34] Bayat M, Aghili M (2014). Transmission of Religious Concepts Ethics with Human Nobility Approach. Iranian Journal of the Knowledge Studies in the Islamic University..

[35] Naghibi A (2012). Elements of human dignity in the Qur’an and practuce of the fourteen infallibles (AS). Seraje Monir.

[36] Naghibi A (2012). Elements of human dignity in the Qur’an and practuce of the fourteen infallibles (AS). Seraje Monir..

[37] Suranari H (2010). The globalization of economy and the implications of the principle of human dignity in the development right. Allameh..

[38] Jafari Tabrizi MT (1999). A Comparative Study of Universal Human Rights: From the Viewpoints of Islam and the West.

[39] Amid Zanjani AA, Tavakoli MM (2008). Innate dignity of human and Islamic human rights. Law Quarterly. Journal of Faculty of Law and Political Science.

[40] Rahiminejad I (2012). The Islamic view on human dignity. Marifat-i Hoghoughi..

[41] Ibrahimzadeh Amoli A (2008). Human in the perspective of Islam and Humanism. Qabasat..

[42] Afzali MA (2010). Human dignity and euthanasia in Islamic ethics. J Mazandaran University Medical Sciences..

[43] Ibn Manzur M (1993). [Lisan al-arab].

[44] Majlisi MB (2000). [Bihar al-Anwar].

[45] Alidoost A, Ebrahimirad M (2011). Study of the rule of control and its scope. Islamic Law..

[46] Taheri H (2015). [Ghwaed Feghh].

[47] Mohaghegh Damad SM (1995). The rules of Islamic jurisprudence.

[48] Majlisi MB (1982). [Bihar al-Anwar].

[49] Horr Ameli M [Wasail al-Shia].

[50] Mohaqeq Karaki ABM, Qom (1993). [Jamea Al-Maqased Fi Sharhe Al-Qavaed].

[51] Najafi MH (1983). [Jawaher al-kalam fi sharh sharaea al-eslam].

[52] Tusi M (1986). [Al-Khilaf].

[53] Abedi Sarasiya A (2017). The Principle of sovereignty and its role in correcting the newly-emerged contracts (with special reference to Imam Khomeini’s viewpoints). Pajouheshnameh Matin..

[54] Momen Qomi M (1994). [Kalimat Sadideh Fi Masail Jadideh].

[55] Kaldjian LC, Wu BJ, Jekel JF, Kaldjian EP, Duffy TP (1999). Insertion of femoral-vein catheters for practice by medical house officers during cardiopulmonary resuscitation. N Engl J Med.

[56] Rathor MY, Rani MF, Shah AS, Leman WI, Akter SF, Omar AM (2011). The principle of autonomy as related to personal decision making concerning health and research from an ‘Islamic Viewpoint’. JIMA..

[57] Potter J (2015). Does the Iranian model of kidney donation compensation work as an ethical global model?. Online Journal of Health Ethics..

[58] Mohamadrezaei M (2011). A critical study on Kant`s view of Human autonomy and freedom. Religious Anthropology..

[59] Shariati S, Ensafi Mehrbani S (2014). An Investigation into “compulsion “and “free will” in Islamic school and its educational implications. Qur'anic Knowledge..

[60] Mesbah Yazdi MT (2014). Theology, Cosmology, Anthropology (Meshkat).

[61] Mesbah Yazdi MT (2009). Teaching Philosophy.

[62] Tabatabai MH (1971). [Al-Mizan Fi Tafsir Al-Qur'an].

[63] Mohaghegh Damad SM (2013). The Rules of Islamic Jurisprudence: Civil Section.

[64] Mohammady A (2010). Rules of Islamic law.

[65] Kulayni M (1986). [Al-kafi].

[66] Horr Ameli MBH (1988). [Wasail Al- Shia].

[67] Zeraat A (2009). Civil Jurisprudence Rules.

[68] Karimi A, Shabani Kandsari H (2015). The logical relationship between no loss principle in Islamic jurisprudence and the principle of abuse of rights in western legal systems. Quarterly Journal of Comparative Studies on Islamic and Western law..

[69] Alidoust A Islamic Jurisprudence (Fegh) and Expediency.

[70] Jafari Langroudi MJ (1996). Law Terminology.

[71] Mohaghegh Damad SM (2011). The Rules of Islamic Jurisprudence penal section.

[72] Farzaneh A (2012). The role of necessity and urgency rule in the legitimacy of exerting non-proprietorship by the government. The Quarterly Journal of Jurisprudence & History of Civilization..

[73] Safaei S, Abbasi M (2015). No harm principle in Islamic Jurisprudence and law and its usage in biomedical Jurisprudence. Journal of Bioethics..

[74] Bahrami Ahmadi H (2009). Islamic Jurisprudence Rules.

[75] Qomi Sadugh M, Ali Akbar Ghaffari (1983). [Al-Khisal].

[76] Torayhi F (1995). [Majma al-Bahrayn].

[77] Mohaghegh Hilli J (1982). [Maareja al-Usul].

[78] Pirhayati H (2015). The place rule of expedient in Iranian criminal law. Judgment..

[79] Farhangi MH (2006). The Relation between jurisprudence and common legal sources and essentials of sovereignty. Philosophical Investigations..

[80] Salarzaei AH, Janjan F (2012). Criminal policy originated from the Holly Quran. Crime Prevention Studies Quarterly..

